# Sexual signals reflect telomere dynamics in a wild bird

**DOI:** 10.1002/ece3.2948

**Published:** 2017-04-04

**Authors:** Conor Claverie Taff, Corey R. Freeman‐Gallant

**Affiliations:** ^1^ Lab of OrnithologyCornell UniversityIthacaNYUSA; ^2^ Department of BiologySkidmore CollegeSaratoga SpringsNYUSA

**Keywords:** honest signaling, senescence, trade‐offs

## Abstract

Telomere dynamics in natural populations have been linked to survival, reproduction, and energetic investment. Given their putative role in mediating life‐history trade‐offs, telomeres are also a likely candidate for maintaining honesty in sexually selected signals; few studies to date, however, have demonstrated a correlation between sexual signals and telomere dynamics. Here, we show that plumage coloration in male common yellowthroats (*Geothlypis trichas*) is correlated with both relative telomere length and with the rate of telomere loss between years. Elevated antioxidant capacity is also associated with reduced telomere loss, but only among older males. Previous work in this population has demonstrated that males with brighter plumage are in better condition, have higher reproductive success, and are more likely to survive over winter. Thus, the signal attribute associated with mate choice in this system also conveys reliable information about telomere dynamics. At present, it is unclear whether telomere maintenance plays a causal role in maintaining signal honesty or whether the correlation arises due to underlying variation in individual resources or genotypes. We suggest that subsequent work should consider the possibility that fundamental trade‐offs between signal investment and cell‐level processes that influence aging and reproductive senescence may provide a foundation for understanding the maintenance of sexual signal honesty.

## 1 Introduction

Telomeres have long been of interest in human medical research (Blasco, 2005), but it is only in the past 15 years that evolutionary ecologists have begun to study telomere dynamics in wild populations (Monaghan & Haussmann, 2006; Nussey et al., 2014). Telomeres are regions of repeated base pairs that cap the ends of eukaryotic chromosomes and function in meiosis, DNA repair, protection of genetic material, and cellular senescence (Monaghan & Haussmann, 2006). A critical feature of telomeres is that they can both (1) be influenced by organismal behavior that modifies the cellular environment (Kotrschal, Ilmonen, & Penn, 2007; Nettle et al., 2015; Salmon, Nilsson, Nord, Bensch, & Isaksson, 2016) and (2) act as functional constraints on performance by driving apoptosis and, ultimately, senescence at the level of the whole organism (Boonekamp, Mulder, Salomons, Dijkstra, & Verhulst, 2014; Monaghan & Haussmann, 2006). Thus, telomeres offer a proximate explanation for trade‐offs that govern the evolution of life‐history strategies including those related to mate choice and parental investment (Angelier, Vleck, Holberton, & Marra, 2013; Bauch, Becker, & Verhulst, 2013; Bebbington et al., 2016; Bize, Criscuolo, Metcalfe, Nasir, & Monaghan, 2009; Geiger et al., 2012; Pauliny, Wagner, Augustin, Szep, & Blomqvist, 2006; Sudyka et al., 2014).

Understanding how signal honesty is maintained has long been a goal of evolutionary ecologists (Alonso‐Alvarez, Bertrand, Faivre, Chastel, & Sorci, 2007; Folstad & Karter, 1992; Hamilton & Zuk, 1982; Hill, 2014; Zahavi, 1975). One long‐standing idea is that signals may remain honest when they are costly to produce (Zahavi, 1975). Clear support for this hypothesis depends on identifying the mechanism(s) that link signal production to organismal costs. For example, signal production might change resistance to parasites (Hamilton & Zuk, 1982), alter immunocompetence (Folstad & Karter, 1992), or be directly linked to processes such as cellular respiration (Hill, 2014). Recent attention has focused on the way that signal investment alters oxidative metabolism by increasing the production of reactive oxygen species (ROS) or by decreasing oxidative defenses (Alonso‐Alvarez et al., 2007). Oxidative damage is thought to be the largest driver of telomere erosion (Monaghan & Haussmann, 2006); thus, we propose that sexual signals might reflect telomere dynamics if signal production incurs a cost (cellular senescence) in proportion to investment (Giraudeau et al., 2016). Our hypothesis need not be an alternative to other honesty mechanisms; rather, telomere erosion may function as the “currency” through which costs incurred by parasite defenses, immune activation, and oxidative damage are “paid.”

We studied telomere dynamics, sexual signals, and oxidative metabolism in common yellowthroats (*Geothlypis trichas*). Common yellowthroats possess both a melanin‐based black facial mask and a UV to yellow, carotenoid‐based bib, both of which are targets of sexual selection in our population (Freeman‐Gallant et al., 2011; Taff, Freeman‐Gallant, Dunn, & Whittingham, 2013). Among males in their first breeding season, bib traits are positively associated with body condition, reduced oxidative damage to DNA, and survivorship and are under strong directional selection with respect to social mating success, the fitness component that generates the greatest opportunity for sexual selection in this age group (Freeman‐Gallant et al., 2011, 2011). By contrast, among older males, the information content of some bib traits is attenuated or reversed, and selection occurs primarily through extra‐pair mating success (Freeman‐Gallant et al., 2011; Taff et al., 2013). Here, we show that the coloration of the yellow bib in a male's first breeding season is correlated with both relative telomere length and with the rate of subsequent telomere loss. These findings suggest that females evaluating bib coloration gain information about the telomere dynamics of potential mates and that telomere dynamics may contribute to the maintenance of honesty in this signal.

## 2 Materials and Methods

We studied 59 adult male common yellowthroats at two field sites in Saratoga County, NY, from 2008 to 2012. These males were present in our study site for 1–5 years (mean ± *SD* = 2.0 ± 1.1). All adult common yellowthroats breeding at these field sites have been captured and banded every year since 2005. Territory fidelity for adult males at our site is extremely high and 97% of returning males settle within 400 m of their previous year's territory (Taff et al., 2013); thus, we used years since banding as a proxy for age (as in Freeman‐Gallant et al., 2011). Although presence on the breeding grounds has been monitored at our site in every year since 2005, the samples required to measure telomeres, total antioxidant capacity, and oxidative damage were not collected until 2008 and were not always available for all males even after 2008. Thus, the main analyses investigating between year changes in telomere length include a total of 47 comparisons of consecutive years within males. Of those comparisons, 27 represent a transition from a male's first to second breeding season, while the remaining 20 represent transitions between older age classes (ages 2–3 *n* = 13; ages 3–4 *n* = 5; ages 4–5 *n* = 2). Due to both the difference in patterns of sexual selection for first‐time breeders (Freeman‐Gallant et al., 2011) and the small sample sizes for year‐to‐year transitions among older birds, we grouped year‐to‐year transitions after the second breeding season into one age category in our analyses.

Each season, we captured males in mist nets and conducted detailed censuses daily. At capture, we took photographs, feathers, and blood samples (Freeman‐Gallant et al., 2011; Taff et al., 2013). We used photographs to measure the size of the mask and bib (Freeman‐Gallant et al., 2011). We measured the UV brightness, yellow brightness, and carotenoid chroma of feathers plucked from the bib with an Ocean Optics 2000 UV‐VIS spectrophotometer (details in Freeman‐Gallant et al., 2011; Taff et al., 2013). Plumage measures were standardized and corrected for population differences across years (Freeman‐Gallant et al., 2011). Whole blood collected for telomere and oxidative damage assays was added to ice‐cold buffer in the field (10% DMSO, 90% newborn calf serum) and later cryopreserved at −80°C (Freeman‐Gallant et al., 2011). A separate blood sample was stored in a cooler until returning to the laboratory, where it was centrifuged and plasma was stored at −20°C until total antioxidant capacity (TAC) was measured at the end of each breeding season (Taff & Freeman‐Gallant, 2014).

### 2.1 Telomere assay

Samples that had been cryopreserved for telomere analysis from 2008 to 2012 were thawed in June 2013, and DNA was extracted using DNeasy Blood and Tissue kits (Qiagen) following the manufacturer's protocol. We verified the integrity of our extracted DNA samples by running each sample on a 2% agarose gel for 100 V‐hrs; in all cases, the DNA formed a single sharp band with high molecular mass, indicating little DNA degradation. We checked the concentration and purity of our DNA using a NanoDrop 2000 Spectrophotometer (ThermoScientific) and further purified samples showing an absorbance ratio (260/280 nm) less than 1.7 using a wash consisting of 100% ethanol and a 1:20 dilution of 5M‐NaCl:sample.

Telomeres were measured using a quantitative real‐time PCR method that has been validated in birds (Cawthon, 2002; Criscuolo et al., 2009). We amplified a single copy control gene (GAPDH: glyceraldehyde‐3‐phosphate dehydrogenase) using the primers GAPDH‐F (5′‐TTGACCACTGTCCATGCCATCAC‐3′) and GAPDH‐R (5′‐TCCAGACGGCAGGTCAGGTC‐3′). Telomere repeats (TTAGG)_*n*_ were amplified using the primers Tel1b (5′‐CGGTTTGTTTGGGTTTGGGTTTGGGTTTGGGTTTGGGTT‐3′) and Tel2b (5′‐GGCTTGCCTTACCCTTACCCTTACCCTTACCCTTACCCT‐3′). Each of our reactions consisted of 20 ng of sample DNA, 12.5 μl of *Power* SYBR^®^ Green PCR Master Mix (Life Technologies, Carlsbad, CA, USA), and forward and reverse primers for either GAPDH or telomere amplification. Both telomere and GAPDH forward and reverse primers were used at a concentration of 50 nmol/L in a final volume of 25 μl per reaction. We ran our reactions on a StepOnePlus^™^ qPCR system (Applied Biosystems) and followed Barrett et al. (2012) in including our telomere and GAPDH reactions in different wells on the same plate (Barrett et al., 2012). Running both reactions on one plate reduced amplification efficiency for each target because our qPCR conditions were intermediate between the two target optima, but improved accuracy by ensuring that telomere and GAPDH samples from each individual were run at the same time and with the same working solution of reagents. Our qPCR conditions consisted of an initial 15 min at 95°C, followed by 40 amplification cycles (15 s at 95°C, 30 s at 58°C, and 30 s at 72°C), and a melt curve from 60 to 95°C with steps of 0.3°. Amplification efficiencies were calculated from the standard curve of pooled DNA samples in each plate and were 81.0 ± 3.9% for telomere targets and 93.5 ± 4.6% for GAPDH targets.

Traditional cycle‐threshold (*C*_t_) methods for scoring qPCR results are subject to errors when the assumption of uniform amplification efficiencies across wells is not met (e.g., when immunoglobulins inhibit PCR reactions in some wells). Thus, we followed the *C*_y0_ method described by Guescini, Sisti, Rocchi, Stocchi, and Stocchi (2008) to score our telomere amplification data. This method has the advantage of not depending on user defined threshold levels and produces unbiased estimates of target DNA even when amplification efficiency varies between wells. We used the amplification data from each well to fit a nonlinear, five‐parameter Richards function:

$$F_{x}=\frac{F_{\max}}{{\left[1+\left(-\frac{1}{b}\left(x-c\right)\right)\right]}^{d}}+F_{b}$$

where *x* is the cycle, *F*_*x*_ is the fluorescence at cycle *x*,* c* is the turning point in the fitted curve, *d* is the Richards coefficient, *F*_*b*_ is the background fluorescence, and *F*_max_ is the maximum fluorescence. Using the parameters from this fitted model, we were able to calculate a *C*_y0_ value for each well as follows:

$$C_{y0}=c+b\ln d-b\left(\frac{d+1}{d}\right)\left[1-\frac{F_{b}}{F_{\max}}{\left(\frac{d+1}{d}\right)}^{d}\right]$$

Our calculated *C*_y0_ values were similar to the *C*_t_ values output by the standard qPCR platform software, indicating that amplification was generally constant across wells (linear regression of TRQ values derived from *C*_t_ versus *C*_y0_ method: *n* = 109, *R* ^2^ = .83, *p* < .0001). We analyzed TRQ values derived from the *C*_y0_ method, but results were qualitatively similar with TRQ values derived from the *C*_t_ method.

Melt curves for each of our final runs showed high specificity of target amplification. Telomere and GAPDH products had similar melt temperatures (75.8 ± 0.1 and 75.8 ± 0.1, respectively), but telomeres had a wider melt peak, as expected given the variable length of telomere amplifications. Our negative controls never showed amplification until very late (negative telomeres: >29 cycles; negative GAPDH: >38 cycles), while telomere and GAPDH samples showed detectable amplification at 13 ± 0.6 and 23.6 ± 0.3 cycles, respectively. Thus, any amplification in negative controls was >13 cycles after target amplification; the high melt temperatures of these products (77.4 ± 0.4 for negative telomeres and 79.7 ± 0.7 for negative GAPDH) suggest that this amplification was the result of primer‐dimerization late in the PCR process.

Each of our 96‐well plates consisted of negative controls for GAPDH and telomeres, a “golden” sample, five standard dilutions, and the target samples from nine birds. Each of these reactions was run in triplicate, and the three wells were averaged to arrive at a final measurement. Our assay did not allow us to estimate the absolute number of telomere repeats in each male. Rather, we measured the amount of telomere repeats relative to standards that allowed for comparisons between individuals in our study. Unknown sample amplifications were first compared to a standard curve made from a pooled sample of DNA from eight different yellowthroats and diluted to five concentrations ranging from 2.5 to 40 ng of template; amplification of our standards was consistent across plates, with *R* ^2^ values of 0.99 ± 0.01 and 0.99 ± <0.01 for telomere and GAPDH standard curves, respectively. After plotting unknown samples on the standard curves, telomere quantification was standardized within each male by dividing telomere amplification by the control gene GAPDH. Finally, we divided the resulting values for each male by the value for the “golden” standard sample run in the same plate. The intraplate coefficient of variation for TRQ measures was 7.4%, and repeatability was 0.90. The interplate coefficient of variation for the “golden” standard before normalizing was 15%. All measures were standardized within plates and comparisons made internally (i.e., all samples from multiple years for the same bird were always run on the same plate).

### 2.2 Oxidative metabolism assays

We measured two aspects of oxidative metabolism from each of our birds. First, we quantified oxidative damage to the DNA in red blood cells using Trevigen CometSlides (Gaithersburg, MD, USA) as described in previously published work on this population (Freeman‐Gallant et al., 2011). For this assay, we thawed cryopreserved cells and suspended them on agarose slides, lysed the cells, denatured the DNA, and then subjected slides to electrophoresis for 10 min at 35 V. Slides were then stained with SYBR Green and digitally imaged. We used the program Comet Score version 1.5 to quantify the relative amount of DNA in the “tail” (damaged) vs. the “head” (intact) of each cell. We averaged this percentage across ~150 cells for each sample to arrive at a final measure of oxidative damage for each individual; this percentage was square‐root arcsine transformed prior to analysis.

Second, we measured the total antioxidant capacity (TAC) of plasma from each of our birds using a commercially available microplate kit (Cayman Chemical, Ann Arbor, MI, USA) and following the manufacturer's recommended protocol and dilutions (as in Taff & Freeman‐Gallant, 2014). This TAC measure quantifies the sum of endogenous and food‐derived antioxidants in plasma. This general measure gives an overall indication of ability to protect against the damaging effects of reactive oxygen or nitrogen radicals, but does not specify which particular antioxidants are represented in the plasma.

### 2.3 Statistical analysis

To test for a relationship between coloration and telomere erosion, we fit a linear mixed model (LMM) with the change in relative telomere length from year *N* to *N* + 1 (ΔTRQ)—corrected for expected regression to the mean (following instructions in Kelly & Price, 2005)—as the response variable. This model included all males with at least two consecutive years of data with a random effect for male identity. Predictors included year *N* bib coloration (UV brightness, yellow brightness, and carotenoid chroma), bib size, mask size, and relative telomere length. We also included oxidative damage and TAC as predictors in this model, and for these values, we averaged measurements over year *N* and *N* + 1; in a few cases, these measures were only available from 1 year and we used the single value as our predictor. Finally, we included a two‐level categorical predictor for male age that differentiated pairs of years in which year *N* represented a male's first breeding season from those of older males. We checked for two‐way interactions between male age and each predictor by adding these interactions one at a time; only significant interactions were retained. Significance was assessed with likelihood ratio tests comparing the full model to reduced models that excluded one predictor at a time. After this approach indicated that bib UV brightness was related to the rate of telomere loss, we also tested for simple correlations between year *N* UV brightness and telomere length in years *N*,* N* + 1, and *N* + 2. For this analysis, we included only a subset of males that were initially sampled as first‐time breeders at our site. Finally, we tested for relationships between telomere length or rate of telomere erosion and survival by fitting two GLMMs; the first of these models include telomere length in year *N* as a predictor and survival to year *N* + 1 as a response, while the second included change in telomere length from year *N* to *N* + 1 as a predictor and survival to year *N* + 2 as a response.

## 3 Results

**Figure 1:**
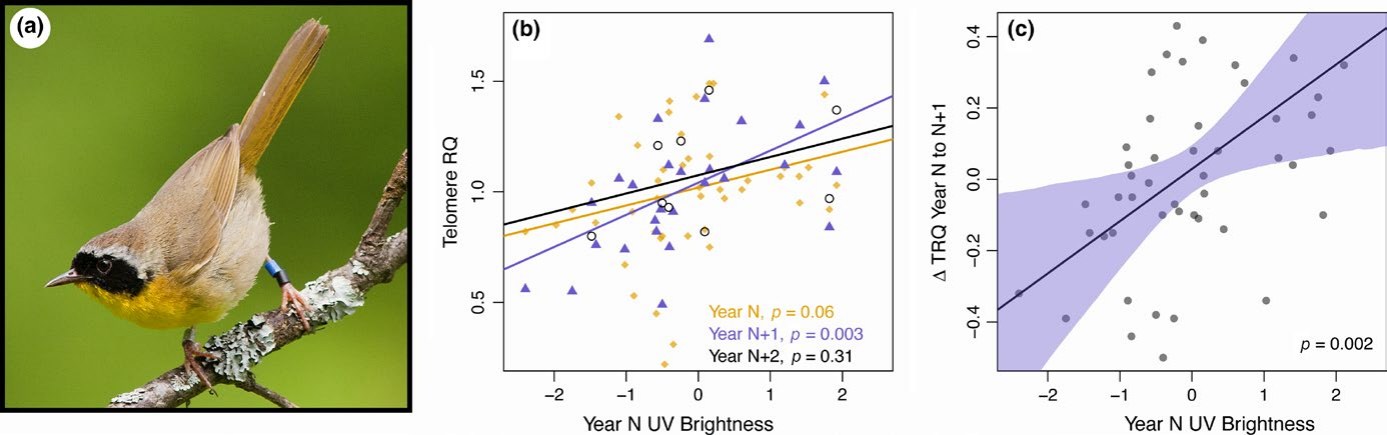
(a) Male common yellowthroat displaying the black facial mask and UV‐yellow bib. (b) Relationship between standardized year *N* UV brightness (positive values indicate brighter males) and relative telomere length in year *N* (orange diamonds, *n* = 47), year *N* + 1 (blue triangles, *n* = 27), and year *N* + 2 (open black circles, *n* = 9) for males that were initially sampled as first‐time breeders. (c) Model predicted relationship between standardized year *N* UV brightness (positive values indicate brighter males) and ΔTRQ (negative values indicate faster telomere loss) for all males with two or more consecutive years of data. Shaded region illustrates the 95% confidence interval based on 1,000,000 samples drawn from the fit model in Table 1 with all predictors except for UV brightness held at mean values

**Figure 2:**
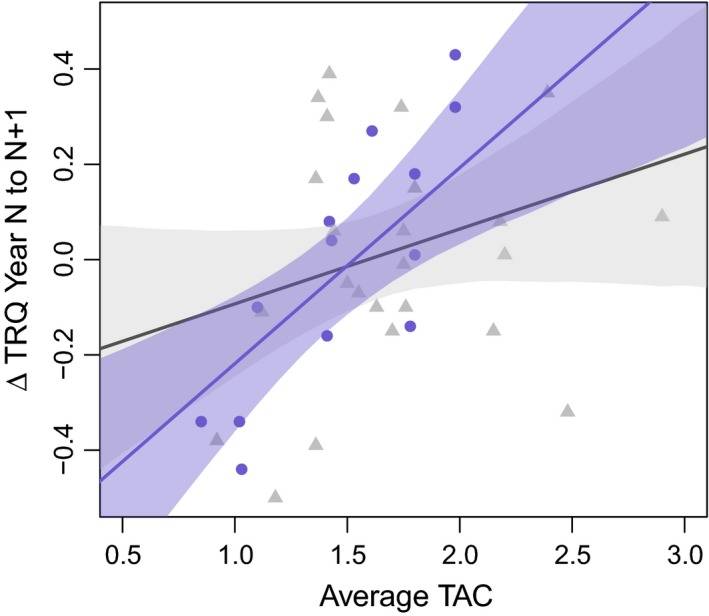
Relationship between average TAC across years *N* and *N* + 1 and ΔTRQ by male age. Points show the raw data for males in their first breeding season (gray triangles) and older males (blue circles). Fit lines and confidence intervals are based on the full model in Table 1 with all covariates except for age and TAC held at mean values. The model predicted relationship and 95% confidence interval is illustrated separately for males in their first breeding season (gray line and shading) and older males (blue line and shading)

In an LMM including all males with complete physiology and signaling data from two (*n* = 20) or three (*n* = 9) consecutive years, males with higher UV brightness experienced less telomere erosion from year *N* to *N* + 1 (Figure 1c; Table 1: *p* = .002). In the same model, higher levels of TAC were associated with reduced rates of telomere erosion, but only among older males (Figure 2; Table 1: interaction between age and TAC, *p* = .02). Among birds in their first breeding season, there was a marginally nonsignificant positive relationship between year *N* UV brightness and year *N* TRQ (Figure 1b; year *N*:* r* = .27, *df* = 45, *t* = 1.9, *p* = .06), a positive relationship between year *N* UV brightness and year *N* + 1 TRQ (year *N* + 1: *r* = .55, *df* = 25, *p* = .003) and no relationship between year *N* UV brightness and year *N* + 2 TRQ, albeit with a much smaller sample size (year *N* + 2: *r* = .38, *df* = 7, *p* = .31).

**Table 1:** LMM with ΔTRQ as the response variable and year *N* TRQ, year *N* ornamentation, average DNA damage, average TAC, and male age as predictors. Model includes 38 observations of between year changes for 29 males with male identity as a random effect. Two‐way interactions are retained if the effect was significant. Significance was assessed by likelihood ratio tests comparing full and reduced models

Predictor	Estimate	*SE*	χ^2^	*p*
Intercept	−0.19	0.74		
Year *N* telomere RQ	−0.11	0.11	0.2	.64
Avg. DNA damage	0.01	0.01	1.4	.24
Avg. antioxidant capacity (TAC)	−0.10	0.19	8.8	.003
Year *N* UV brightness	0.15	0.06	9.3	.002
Year *N* yellow brightness	−0.01	0.06	0.2	.66
Year *N* carotenoid chroma	0.07	0.05	2.5	.12
Year *N* bib size	0.01	0.05	0.1	.75
Year *N* mask size	0.01	0.05	0.0	.99
Male age	−0.38	0.18	0.3	.59
Male Age * Avg. TAC	0.25	0.12	5.6	.02

We did not find any evidence that telomeres consistently shortened with age in adult common yellowthroats, either when pooling males (LMM with TRQ as the response, *n* = 109 observations of 59 males; effect of male age: χ^2^ = 0.52, *p* = .47) or when looking at changes within males across years (paired *t* test: *df* = 46, *t* = 0.74, *p* = .46). Finally, we found no evidence that telomere dynamics predicted survival to subsequent years, although there was a nonsignificant trend for individuals with longer telomeres in year *N* to be more likely to survive to year *N* + 1 (binomial GLMM; TRQ in year *N* and survival to year *N* + 1: *n* = 89 observations of 56 males, χ^2^ = 2.9, *p* = .09; ΔTRQ and survival to year *N* + 2: *n* = 30 observations of 25 males, χ^2^ = 0.19, *p* = .67).

## 4 Discussion

We found that plumage coloration acts as a reliable indicator of the rate of subsequent telomere erosion. Importantly, the plumage a male displays in the breeding season was acquired the previous year, before migration and, for young males, only several weeks after leaving the nest. Surprisingly, then, our results indicate that a male's plumage can convey information about telomere dynamics 1–2 years later despite the fact that feathers are inert (and their signal attributes fixed) once molt is complete. At present, it is uncertain whether telomere erosion functions directly in maintaining signal honesty or whether telomere erosion and signal expression are correlated due to underlying between‐individual differences in condition or genotype. As with any trade‐off, experimental studies will be needed to conclusively demonstrate causal links (Getty, 1998; Reznick, Nunney, & Tessier, 2000). However, given the well‐established links between—on the one hand—oxidative metabolism, telomere erosion, and senescence (Badás et al., 2015; Haussmann & Heidinger, 2015; Haussmann, Longenecker, Marchetto, Juliano, & Bowden, 2012; Herborn et al., 2014; Kim & Velando, 2015; Monaghan, 2014) and—on the other hand—oxidative metabolism and carotenoid‐based signal expression (Beamonte‐Barrientos, Velando, & Torres, 2014; Hill, Hood, & Huggins, 2009; Simons, Cohen, & Verhulst, 2012), we suggest that trade‐offs between signal investment and telomere maintenance are likely to occur. Regardless of whether telomere dynamics directly influence coloration, it is clear that when females evaluate males on the basis of bib coloration, as we have demonstrated previously in this population (Dunn, Whittingham, Freeman‐Gallant, & Decoste, 2008; Freeman‐Gallant et al., 2011; Taff et al., 2013), they effectively choose mates with more favorable telomere phenotypes.

In some cases, upregulation of oxidative defenses may protect against telomere loss. For example, in Adelie penguins (*Pygoscelis adeliae*), experimental increases in the cost of reproduction result in elevated TAC, but have no effect on telomere loss (Beaulieu, Reichert, Le Maho, Ancel, & Criscuolo, 2011). Similarly, we found that high levels of TAC appear to offer protection against telomere erosion, but only among older males. Thus, younger males may face stricter trade‐offs between oxidative metabolism, telomere erosion, and ultimately signal investment, explaining previously described differences in signal honesty and selection between age classes in our population (Freeman‐Gallant et al., 2011; Taff et al., 2013). Similar examples of context dependent protection of telomeres have been documented in other species (Kim & Velando, 2015; Noguera, Metcalfe, Boner, & Monaghan, 2015).

We did not find evidence that telomeres consistently shortened across years or that rate of telomere erosion predicted survival. It is unclear why we failed to detect these predicted relationships, but much of the previous work on telomeres in natural populations has focused on long‐lived species sampled at larger intervals than a single year (but see Angelier et al., 2013). Males banded as adults at our site are only present for 1.6 ± 1.0 breeding seasons (*n* = 335 Conor C Taff & Corey R Freeman‐Gallant, unpublished data). Thus, spacing samples further apart was not feasible.

Our study is among the first to demonstrate that sexual signals are correlated with telomere dynamics (but see Giraudeau et al., 2016) and the first to demonstrate such a correlation in birds. Clearly, our understanding of the role of telomere dynamics in survival, senescence, and signaling in natural populations is in the early stages. More longitudinal studies are needed that capture the full reproductive and signaling lifetime of individuals. Because both telomeres and ornamentation change with age, experimental studies will be critical to determine the causal relationships that maintain the positive correlation between telomeres and signals. Despite long‐standing interest in discovering mechanistic constraints that maintain the honesty of sexual signals (Folstad & Karter, 1992; Hamilton & Zuk, 1982), progress has been hindered by mixed empirical results and system‐specific patterns. Given the inexorable nature of age‐related trade‐offs, we suggest that incorporating telomere dynamics into studies of sexual signaling may provide a more general mechanistic understanding of signal honesty across taxa.

## Ethical Note

This work was approved by the Institutional Animal Care and Use Committees at Skidmore College (Protocol #69) and UC Davis (protocols #13329 and #16362).

## Data Accessibility

The datasets and code supporting this article have been uploaded to Figshare (DOI: http://doi.org/10.6084/m9.figshare.4793734.v1).

## Author's Contributions

CCT and CFG each contributed to study design, data collection, analysis, and writing. Both authors approved the final version and are accountable for the content.

## Conflict of Interest

None declared.
